# No effect of age, gender and total intracranial volume on brainstem MR planimetric measurements

**DOI:** 10.1007/s00330-019-06504-1

**Published:** 2020-01-17

**Authors:** Stephanie Mangesius, Anna Hussl, Susanne Tagwercher, Eva Reiter, Christoph Müller, Lukas Lenhart, Florian Krismer, Philipp Mahlknecht, Michael Schocke, Elke R. Gizewski, Werner Poewe, Klaus Seppi

**Affiliations:** 1 Department of Neuroradiology, Medical University of Innsbruck, Anichstrasse 35, 6020 Innsbruck, Austria; 2 Department of Neurology, Medical University of Innsbruck, Anichstrasse 35, 6020 Innsbruck, Austria; 3 Neuroimaging Core Facility, Medical University of Innsbruck, Anichstrasse 35, 6020 Innsbruck, Austria

**Keywords:** Magnetic resonance imaging, Parkinsonian disorders, Diagnosis, differential, Age factors, Sex factors

## Abstract

**Objectives:**

MR planimetry of brainstem structures can be helpful for the discrimination of Parkinsonian syndromes. It has been suggested that ageing might influence brainstem MR measurements assessed by MR planimetry, while effects of gender and total intracranial volume (TIV) have not been assessed so far. The aim of this study was to evaluate age, gender and TIV effects on brainstem MR planimetric measures.

**Methods:**

Brainstem MR planimetric measures of diameters (midbrain, pons, middle and superior cerebellar peduncle) and areas (pons and midbrain), the derived ratios, and the magnetic resonance Parkinsonism index (MRPI) were assessed on 1.5-T MR images in a large cohort of 97 healthy controls and analysed for the influence of age, gender and TIV with univariate and multivariate linear models.

**Results:**

Neither gender nor age effects on planimetric measurements were observed in the population relevant for the differential diagnosis of neurodegenerative Parkinsonism, aged 50 to 80 years, except for single area-derived measurements, with gender effects on pontine area (*p* = 0.013) and age effects on midbrain area (*p* = 0.037). Results were similar upon inclusion of the TIV in the analyses.

**Conclusions:**

There is no need to correct for age, gender or TIV when using brainstem-derived MR planimetric measurements in the differential diagnosis of neurodegenerative Parkinsonism.

**Key Points:**

*• There were no gender effects on single or combined imaging measurements of the brainstem in the population aged 50 to 80 years, the age range relevant for the differential diagnosis of neurodegenerative Parkinsonism (except for pontine area).*

*• There were no age effects on single or combined imaging measurements of the brainstem in the population aged 50 to 80 years, the age range relevant for the differential diagnosis of neurodegenerative Parkinsonism (except for midbrain area).*

*• There is no need for age- or gender-specific cut-offs for the relevant age group.*

**Electronic supplementary material:**

The online version of this article (10.1007/s00330-019-06504-1) contains supplementary material, which is available to authorized users.

## Introduction

Magnetic resonance (MR) planimetry can assist in the differential diagnosis of progressive supranuclear palsy (PSP) from non-PSP neurodegenerative Parkinsonism [1–5].

While structural changes of the brain regarding decreased brain tissue size and increased brain cerebrospinal fluid volume during ageing have been well-established [6–8], studies investigating age effects on brainstem structures are limited. Negative correlation between midbrain volume and ageing has been shown in several MR studies [9–11]. Moreover, a recent study by Morelli et al. suggested influence of ageing on brainstem-derived measures as pontine area, midbrain area, middle cerebellar peduncle diameter (MCP_d_) and midbrain-to-pons area ratio (*M*_A_/*P*_A_) in PD patients, and on midsagittal area of the midbrain and the *M*_A_/*P*_A_ in healthy controls [12].

Since studies on gender effects have focused on grey and white matter thickness, surface, distribution and integrity in general, effects of gender on the whole brain are well known [13–15], whereas evidence for gender-specific effects of the brainstem volumes remain conflicting [13–15].

As brainstem planimetry is a simple and fast tool to assist for the discrimination of PSP from non-PSP neurodegenerative Parkinsonism with growing interest in the research community over the past decade [1, 3, 16–21], the aim of this study was to explore age- and gender-related effects on brainstem MR planimetric measures in healthy controls.

## Materials and methods

### Study population

Our study involved 97 healthy controls aged 30 to 80 years (30–40 years (*n* = 6); 40–50 years (*n* = 5); 50–60 years (*n* = 13); 60–70 years (*n* = 58), 70–81 years (*n* = 15)). Healthy controls were without history of neurological or psychiatric disease and had normal neurological examinations. Evidence of vascular lesions, including lacunae or infarctions in the midbrain or basal ganglia, as evaluated with routine MRI sequences, was excluded by experienced neuroradiologists.

The healthy controls were recruited as part of three different studies.

All participants provided written informed consent before participating in this study, which was approved by the local Ethics Committee of the Medical University Innsbruck.

### Magnetic resonance imaging protocol and image analysis

High-resolution MR images of all subjects were acquired on a 1.5-T scanner (Magnetom Avanto, Siemens). All participants received a coronal T1-weighted 3-dimensional magnetisation-prepared rapid gradient-echo (3D-MPRAGE) sequence with a TR of 1600 ms, a TE of 3.44 ms, a slice thickness of 1.2 mm, a matrix of 256 × 224 pixels and a field of view of 220 × 192 mm.

**Fig. 1:**
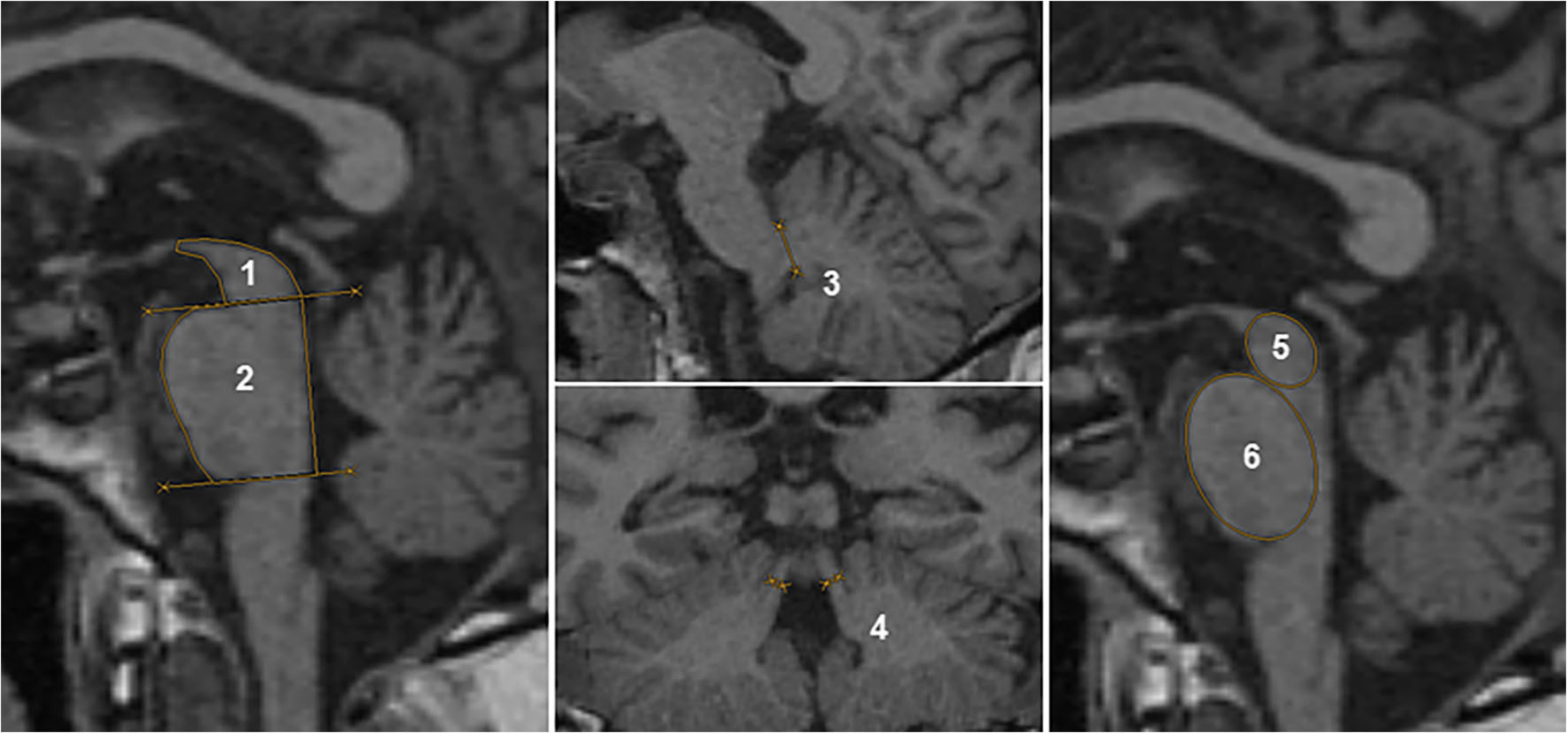
MR planimetric measurements. The midbrain area was depicted between the first line (passing through the superior pontine notch and the inferior edge of the quadrigeminal plate) and the trace of the midbrain tegmentum (1), while the pontine area included the area between the second line (parallel to the first line by passing through the inferior pontine notch) and the anterior and posterior margins of the pons (2). Diameters of left and right middle cerebellar peduncles (MCP_d_) were identified on parasagittal views that best exposed the MCP_d_ between the pons and cerebellum (3). Diameters of left and right superior cerebellar peduncles (SCP_d_) were rated on the first oblique coronal image (parallel to the floor of the fourth ventricle) with inferior colliculi and SCP_d_ separated (4). A mean value for left and right MCP_d_ as well as left and right oblique coronal SCP_d_ was calculated. Elliptical regions of interest were defined over the midbrain (5, without including the collicular plate) and pons (6, excluding the pontine tegmentum). The minor ellipsoid axes, defined as the maximum midsagittal anteroposterior midbrain and pontine diameter, were derived to obtain midbrain diameter and pontine diameter

Midsagittal midbrain area, midsagittal pontine area, middle cerebellar peduncle (MCP_d_) diameter and superior cerebellar peduncle (SCP_d_) diameter were assessed as previously described [1]. Moreover, the midsagittal midbrain diameter and pontine diameter were obtained as recently proposed (Fig. 1) [3]. From these brainstem-derived planimetric measures, the magnetic resonance Parkinsonism index (MRPI), *M*_A_/*P*_A_ and the midbrain-to-pons diameter ratio (*M*_d_/*P*_d_) were calculated.

Planimetric measurements were performed by two experienced raters, all of them blinded to age and gender of the study population. All measurements were repeated by the same investigator and by an independent second investigator. Only the measurements performed by the first rater were included for further calculations.

The estimation of the total intracranial volume (TIV) was conducted using SPM 12 (Statistical Parametric Mapping) while running MATLAB 9.5 (R2018b; MathWorks). T1-weighted images were reoriented and automatically segmented into grey matter (GM), white matter (WM) and cerebrospinal fluid (CSF) with default settings [22]. Native space tissue maps were selected to minimise effects due to spatial normalisation. TIV was calculated as the sum of resulting raw values for GM, WM and CSF.

### Statistical analysis

For evaluation of age and gender effects on the single or combined imaging measurements of the brainstem MRPI, *M*_A_/*P*_A_, *M*_d_/*P*_d_, midbrain diameter, pontine diameter, midbrain area and pontine area in HC, univariate and multivariate analyses were performed. Univariate analyses included calculations of unpaired *t* test for gender, Pearson correlation coefficient *r* for age and performance of univariate general linear models (GLM) for age and gender, including each MR planimetric measurement as dependent variable, gender as factor and age as covariate. Multivariate GLMs were performed with gender as independent variable and age as covariate, including age × gender interaction. Multivariate GLMs were then repeated with gender as independent variable and age as covariate, including age × gender × TIV interaction, to correct for the effect of TIV. In all GLMs, univariate und multivariate, age was used as a continuous variable and not ranked.

Moreover, we repeated the analysis for the population relevant for the differential diagnosis of Parkinsonism, aged 50 to 80 years.

Scatter plots for all single and combined MR planimetric measurements versus age under colour coding for gender including a linear fit model with 95% confidence interval was calculated for raw and TIV-corrected MR planimetric measurements.

Inter- and intrarater variability was calculated using interclass correlation coefficients (ICC, Table 2). All the values were interpreted as follows [23]: values < 0.50 are considered poor; values between 0.50 and 0.75 moderate; values between 0.75 and 0.90 good; and values > 0.90 excellent. Statistical analysis was performed with SPSS v. 22.0 for Windows and GraphPad Prism v. 5.03 for Windows.

## Results

Demographic, clinical and MRI data of participants in the study are shown in Table 1.

**Table 1:** Demographic and MRI data with effects of gender and age on brainstem MR planimetric measurements

				Univariate analysis			Multivariate analysis			
				Gender	Age		Gender	Age	Age × gender	Age × gender × TIV
				*T* (*p* value)^a^	*r* (*p* value)^c^	*F* (*p* value)^b^	*F* (*p* value)^d^	*F* (*p* value)^d^	*F* (*p* value)^d^	*F* (*p* value)^d^
All study participants										
	All (*n* = 97)	Male (*n* = 48)	Female (*n* = 49)							
Age	63.02 ± 10.785	64.47 ± 10.19	61.60 ± 11.26	− 1.312 (0.193)	n.a.	n.a.	n.a.	n.a.	n.a.	n.a.
Midbrain diameter	10.34 ± 0.69	10.42 ± 0.71	10.26 ± 0.68	− 1.140 (0.257)	− 0.328 *(0.001)*	11.491 *(0.001)*	2.829 (0.096)	13.077 *(< 0.001)*	7.270 *(0.001)*	4.635 *(0.012)*
Pontine diameter	17.07 ± 1.14	17.32 ± 1.08	16.83 ± 1.16	− 2.161 *(0.033)*	0.068 (0.505)	0.447 (0.505)	4.322 *(0.040)*	0.157 (0.693)	2.392 (0.097)	3.593 (0.031)
Midbrain area	123.55 ± 14.07	124.02 ± 15.11	123.09 ± 13.10	− 0.325 (0.746)	− 0.393 *(< 0.001)*	17.396 *(< 0.001)*	0.842 (0.361)	18.08 *(< 0.001)*	9.104 *(< 0.001)*	5.224 *(0.007)*
Pontine area	541.215 ± 48.40	552.20 ± 46.87	530.45 ± 47.92	− 2.259 *(0.026)*	− 0.051 (0.619)	0.249 (0.619)	5.497 *(0.021)*	0.672 (0.415)	2.879 (0.061)	2.497 (0.088)
Mean MCP_d_	10.09 ± 0.83	10.19 ± 0.84	10.00 ± 0.81	− 1.131 (0.261)	− 0.276 *(0.006)*	7.821 *(0.006)*	2.457 (0.120)	9.01 *(0.003)*	5.199 *(0.007)*	2.773 (0.068)
Mean SCP_d_	3.68 ± 0.36	3.75 ± 0.39	3.62 ± 0.33	− 1.757 (0.082)	0.215 *(0.035)*	4.589 *(0.035)*	2.273 (0.135)	3.75 (0.056)	3.462 *(0.035)*	4.500 *(0.014)*
*M*_d_/*P*_d_	0.61 ± 0.05	0.60 ± 0.06	0.61 ± 0.05	0.767 (0.445)	− 0.301 *(0.003)*	9.459 *(0.003)*	0.155 (0.695)	8.892 *(0.004)*	4.765 *(0.011)*	5.660 *(0.005)*
*M*_A_/*P*_A_	0.23 ± 0.02	0.22 ± 0.02	0.23 ± 0.02	1.586 (0.116)	− 0.392 *(< 0.001)*	17.220 *(< 0.001)*	1.346 (0.249)	15.72 *(< 0.001)*	9.314 *(< 0.001)*	9.151 *(< 0.001)*
MRPI	12.16 ± 1.64	12.29 ± 1.78	12.03 ± 1.49	− 0.783 (0.435)	−0.031 (0.767)	0.088 (0.767)	0.684 (0.410)	0.164 (0.687)	0.386 (0.681)	0.350 (0.706)
Study participants aged 50 to 80 years										
	All (*n* = 85)	Male (*n* = 43)	Female (*n* = 42)							
Age	66.01 ± 5.82	67.48 ± 4.61	64.50 ± 6.56	− 2.428 *(0.017)*	n.a.	n.a.	n.a.	n.a.	n.a.	n.a.
Midbrain diameter	10.27 ± 0.64	10.32 ± 0.64	10.21 ± 0.64	− 0.755 (0.452)	− 0.035 (0.752)	0.100 (0.752)	0.744 (0.391)	0.279 (0.599)	0.422 (0.657)	0.262 (0.770)
Pontine diameter	17.10 ± 1.17	17.35 ± 1.12	16.84 ± 1.19	− 2.067 *(0.042)*	0.168 (0.123)	2.425 (0.123)	2.962 (0.089)	1.165 (0.284)	2.722 (0.072)	1.855 (0.163)
Midbrain area	121.61 ± 13.35	121.87 ± 14.26	121.34 ± 12.52	− 0.181 (0.857)	− 0.215 *(0.048)*	4.037 *(0.048)*	0.526 (0.470)	4.506 *(0.037)*	2.270 (0.110)	0.403 (0.669)
Pontine area	540.55 ± 50.62	552.60 ± 48.73	528.22 ± 50.09	− 2.275 *(0.026)*	− 0.066 (0.548)	0.364 (0.548)	6.390 *(0.013)*	1.569 (0.214)	3.389 *(0.039)*	2.644 *(0.047)*
Mean MCP_d_	10.02 ± 0.77	10.10 ± 0.79	9.94 ± 0.75	− 0.950 (0.345)	− 0.074 (0.502)	0.454 (0.502)	1.352 (0.248)	0.906 (0.344)	0.904 (0.409)	0.439 (0.646)
Mean SCP_d_	3.71 ± 0.37	3.77 ± 0.39	3.65 ± 0.33	− 1.565 (0.121)	0.192 (0.078)	3.186 (0.078)	1.330 (0.252)	2.048 (0.156)	2.264 (0.110)	1.693 (0.190)
*M*_d_/*P*_d_	0.60 ± 0.05	0.60 ± 0.05	0.61 ± 0.05	1.146 (0.255)	− 0.168 (0.123)	2.425 (0.123)	0.603 (0.440)	1.693 (0.197)	1.508 (0.227)	0.664 (0.517)
*M*_A_/*P*_A_	0.23 ± 0.02	0.22 ± 0.02	0.23 ± 0.02	1.969 (0.052)	− 0.175 (0.109)	2.619 (0.109)	2.582 (0.112)	1.357 (0.248)	2.625 (0.079)	1.951 (0.149)
MRPI	12.16 ± 1.69	12.33 ± 1.81	11.99 ± 1.56	− 0.917 (0.362)	− 0.071 (0.519)	0.420 (0.519)	1.257 (0.266)	0.839 (0.362)	0.839 (0.436)	0.706 (0.497)

Data are given as mean ± SD. Significant effects are marked in italics

^a^Independent samples *t* test

*T*, *t* test; *r*, Pearson correlation coefficient; *r*, univariate and multivariate general linear models; *SCP*_*d*_, superior cerebellar peduncle diameter; *MCP*_*d*_, middle cerebellar peduncle diameter; *M*_*d*_*/P*_*d*_*ratio*, midbrain-to-pons diameter ratio; *M*_*A*_*/P*_*A*_*ratio*, midbrain-to-pons area ratio; *MRPI*, magnetic resonance Parkinsonism index; *n.a.*, not applicable; *SD*, standard deviation

^b^Univariate general linear model (univariate analysis of covariance, ANCOVA)

^c^Pearson correlation

^d^Multivariate general linear model (multivariate analysis of covariance, gender as factor and age as covariate, ANCOVA)

Inter- and intrarater variability was “excellent” with ICCs over 0.90 for all measurements except for the interrater variability of the diameter-based measures which was “good” (Table 2).

**Table 2:** Inter- and intrarater variability assessed by interclass correlation coefficient

Planimetric measurement	Intrarater	Interrater
Midbrain diameter	0.949	0.873
Pontine diameter	0.990	0.845
Midbrain area	1.000	0.992
Pontine area	0.998	0.993
Mean MCP_d_	0.975	0.976
Mean SCP_d_	0.959	0.938
*M*_d_/P_d_	0.955	0.858
*M*_A_/*P*_A_	0.999	0.979
MRPI	0.984	0.993

*SCP*_*d*_, superior cerebellar peduncle diameter; *MCP*_*d*_, middle cerebellar peduncle diameter; *M*_*d*_*/P*_*d*_*ratio*, midbrain-to-pons diameter ratio; *M*_*A*_*/P*_*A*_*ratio*, midbrain-to-pons area ratio; *MRPI*, magnetic resonance Parkinsonism index

All 97 participants were aged 30 to 80 years (mean age, 63.02 ± 10.76 years) and equally gender-distributed (female-to-male ratio = 49:48) including all participants as well as within each age rank. This cohort showed significant gender-related effects on pontine diameter and pontine area in all univariate and multivariate tests performed.

Univariate analyses further revealed age-related effects on midbrain diameter and midbrain area, as well as on MCP_d_, SCP_d_, *M*_d_/*P*_d_ and *M*_A_/*P*_A_, which were confirmed in multivariate analyses, except for SCP_d_ (Table 1).

**Fig. 2:**
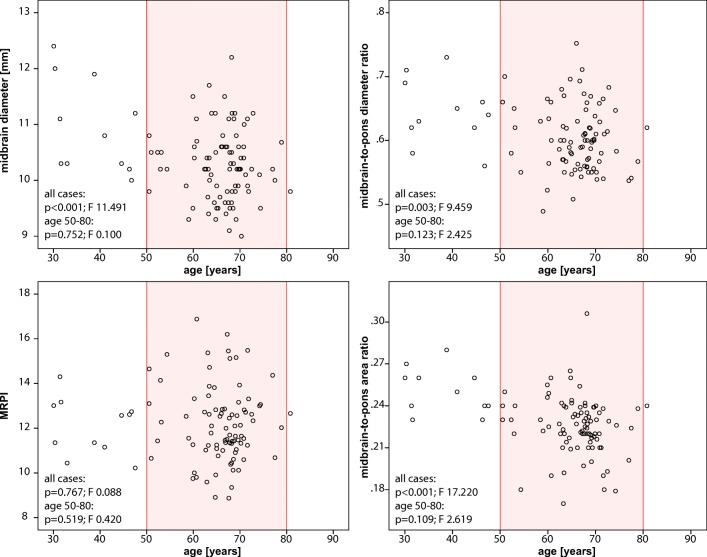
Scatter plots showing the relationship between age and MR measures in healthy population. *MRPI*, magnetic resonance Parkinsonism index

For the cohort aged 50 to 80 years (mean age, 66.01 ± 5.82 years; female-to-male ratio = 42:43), age-related effects were shown by univariate and multivariate analyses for midbrain area only, whereas gender-related effects were significant on both pontine area and diameter with univariate analyses, and only on pontine area with multivariate analyses. There were neither gender- nor age-related effects for M_d_/P_d_, M_A_/P_A_ and MRPI in this cohort (Table 1, Fig. 2). Table 1 includes MR planimetric measurements proven relevant in the differential diagnosis of degenerative Parkinsonism as shown in a recent study [24], and measurements showing gender- or age-related effects.

Results were similar when we included the TIV in the analyses (see Table 1). Only the significant age effects on the MCP_d_ in the whole study cohort vanished when correcting for TIV. Supplementary Fig. 1 further demonstrates all single and combined MR planimetric measurements in relation to age and gender for both, raw measurements and TIV corrected measurements.

## Discussion

In our study, we sought to determine the effects of age and gender on single and combined imaging measurements of the brainstem considered relevant for the differential diagnosis of Parkinsonian syndromes. Our results suggest gender effects on both pontine area and pontine diameter in healthy controls, with male subjects showing larger measurements, and age effects on midbrain diameter, midbrain area, and *M*_d_/*P*_d_ and *M*_A_/*P*_A_.

However, though gender effects on pontine area and age effects on midbrain area were observed, the effect on brainstem ratios as *M*_d_/*P*_d_, *M*_A_/*P*_A_ and MRPI was not confirmed after including only controls aged 50 to 80 years. Moreover, correction of the single and combined imaging measurements of the brainstem for TIV revealed similar results.

A large body of neuroimaging studies have proposed brainstem-derived MR planimetric measures as diagnostic biomarkers for PSP or MSA [16]. To date, it is unclear whether gender and age effects should be considered for these measurements, which would highlight the need for gender- and age-adapted cut-off levels. Gender effects on planimetric measurements have never been investigated so far. To date, there has been one study suggesting age effects on brainstem-derived planimetric measurements in healthy controls, PD and PSP patients [12], not including however gender as an important demographic variable in the analyses, such as false-positive findings cannot be excluded in this study [12].

When analysing the population aged 50 to 80 years, i.e. the age range relevant for the differential diagnosis of neurodegenerative Parkinsonism, neither age nor gender effects on combined brainstem planimetric measures (i.e. *M*_A_/*P*_A_, *M*_d_/*P*_d_ and MRPI) nor midbrain diameter could be detected, suggesting that age and gender effects on combined measurements mainly derive from the younger study population.

Given the large number of healthy participants in this study, the present results are promising and are therefore valid for brainstem planimetric measurements performed in the cohort aged 50 to 80 years. However, further studies are needed to verify the observed gender and age effects on MR planimetric measurements of the brainstem in participants aged 30 to 50 years, which might be of interest for early-onset Parkinsonism and other neurological disease entities, such as e.g. multiple sclerosis.

Therefore, there is no need for age- or gender-specific cut-offs for the age group relevant for the differential diagnosis of neurodegenerative Parkinsonism. It is however unclear if there are effects of disease stages or disease duration on single or combined brainstem MR planimetric measurements as suggested previously in diseased patients [12]. From a clinical point of view, this issue however is not relevant, as differential diagnosis of neurodegenerative Parkinsonism is relevant in the early disease stages only.

MR planimetric measurements were performed as described in the literature [3, 5, 8]. However, none of these studies corrected the brainstem-derived planimetric measures for TIV. If quantitative assessments of regional cerebral atrophy such as brainstem-derived planimetric measures are not corrected for TIV, the effect of age or gender might be compromised by a hidden association with TIV. Therefore, we corrected our analyses for TIV, to exclude an underlying association of reported effects on MR planimetry. To our knowledge, our study is the first to exclude any effect of TIV on brainstem-derived MR planimetric measurements.

A limitation of our study is that MRI images were obtained on 1.5-T scanners only, which potentially might be a source for a methodological bias when being reproduced on 3-T MRI. There is, however, evidence that different scanners produce similar results of quantifiable, infratentorial changes using MRI planimetry on both 1.5-T and 3-T MR images. We therefore conclude that our present results can be transferred without methodological bias to the use of different scanners [25, 26]. Because MR planimetry poses the risk of reproducibility bias, we have assessed inter- and intrarater reliability, which both were high confirming the high inter- and intrarater reliability of MR planimetry–derived measures [3, 12] used for the discrimination of PSP from other forms of Parkinsonism.

In conclusion, our study indicates that there is no need for age- or gender-specific cut-offs of the brainstem-derived MR planimetric measurements used for the differential diagnosis of degenerative Parkinsonism. Moreover, TIV has no effects on these measurements. Therefore, the results of our study have important practical implications for the routine diagnostic work-up of patients with degenerative Parkinsonism when using brainstem-derived MR planimetric measurements as an easy-to-perform procedure.

## Electronic supplementary material

Supplementary Fig. 1 Scatter plots for all single and combined MR planimetric measurements versus age under colour coding for gender (black dots = female; red dots = male subjects) and linear fit model (with 95% confidence interval) for a) raw b) TIV corrected MR planimetric measurements. Abbreviations: SCP_d_ = superior cerebellar peduncle diameter, MCP_d_ = middle cerebellar peduncle diameter; M_d_/P_d_-ratio = midbrain-to-pontine-diameter-ratio; M_A_/P_A_-ratio = midbrain-to-pons-area-ratio; MRPI = magnetic resonance Parkinsonism index. (DOCX 485 kb)

## Abbreviations

3D-MPRAGE: 3-dimensional magnetisation-prepared rapid gradient-echo
CSF: Cerebrospinal fluid
GLM: General linear models
GM: Grey matter
ICC: Interclass correlation coefficients
*M*_A_/*P*_A_: Midbrain-to-pons area ratio
MCP_d_: Middle cerebellar peduncle diameter
*M*_d_/*P*_d_: Midbrain-to-pons diameter ratio
MRPI: Magnetic resonance Parkinsonism index
MSA: Multiple system atrophy
PSP: Progressive supranuclear palsy
SCP_d_: Superior cerebellar peduncle
WM: White matter
