# Supplementary figures and images for: No effect of age, gender and total intracranial volume on brainstem MR planimetric measurements
Source: Eur Radiol. 2020 Jan 17;30(5):2802–8. doi: 10.1007/s00330-019-06504-1 (PMC7160097; doi:10.1007/s00330-019-06504-1)

**Supplementary Materials**


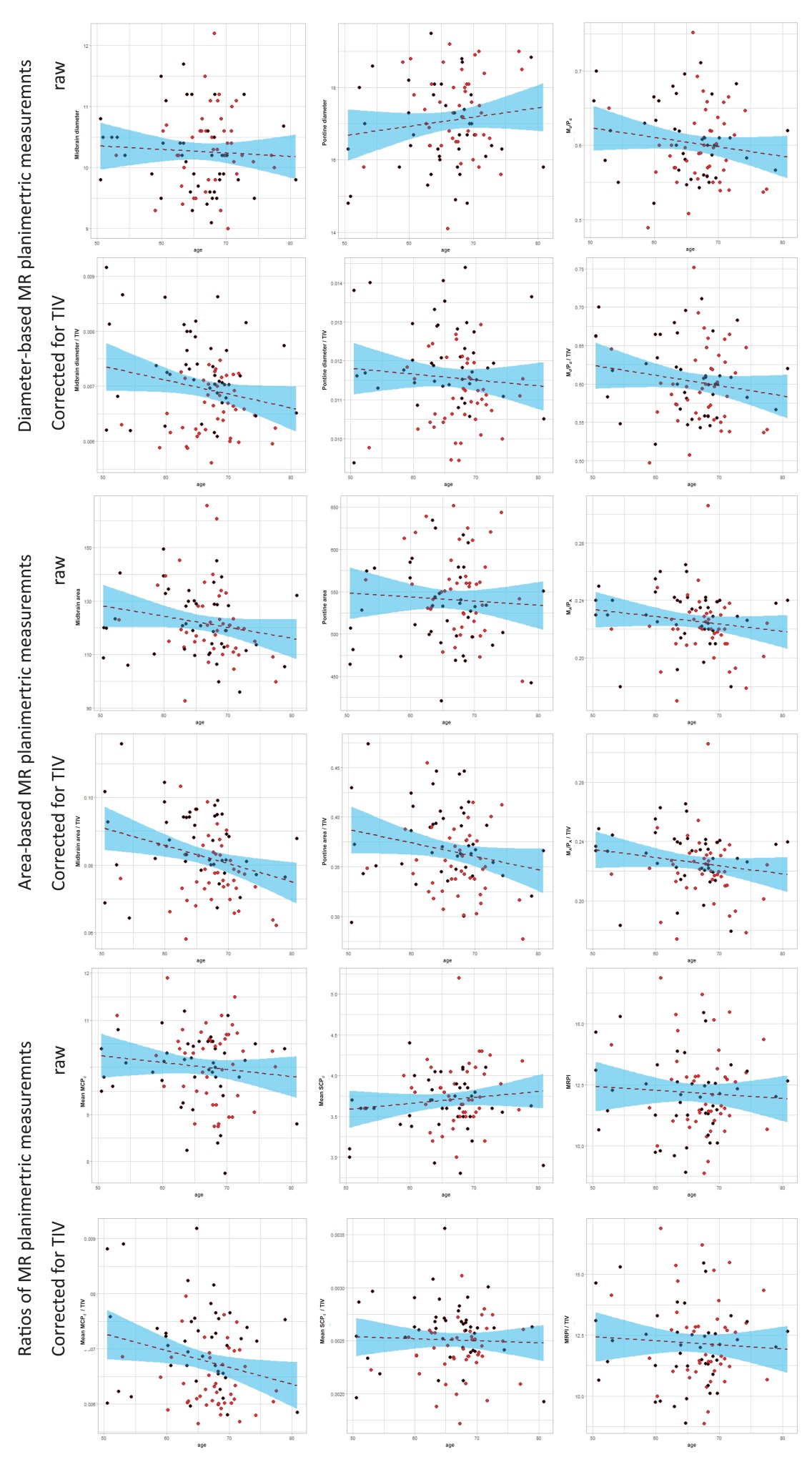

Supplement: Supplementary file 1 — Scatter plots for all single and combined MR planimetric measurements versus age under colour coding for gender (black dots = female; red dots = male subjects) and linear fit model (with 95% confidence interval) for a) raw b) TIV corrected MR planimetric measurements. Abbreviations: SCPd = superior cerebellar peduncle diameter, MCPd = middle cerebellar peduncle diameter; Md/Pd-ratio = midbrain-to-pontine-diameter-ratio; MA/PA-ratio = midbrain-to-pons-area-ratio; MRPI = magnetic resonance Parkinsonism index. (DOCX 485 kb) [file 330_2019_6504_MOESM1_ESM.docx]
